# Outbreak of colistin resistant, carbapenemase (*bla*_NDM_, *bla*_OXA-232_) producing *Klebsiella pneumoniae* causing blood stream infection among neonates at a tertiary care hospital in India

**DOI:** 10.3389/fcimb.2023.1051020

**Published:** 2023-02-01

**Authors:** Ashutosh Pathak, Nidhi Tejan, Akanksha Dubey, Radha Chauhan, Nida Fatima, Jyoti, Sushma Singh, Sahil Bhayana, Chinmoy Sahu

**Affiliations:** ^1^ Department of Microbiology, Sanjay Gandhi Post Graduate Institute of Medical Sciences, Lucknow, India; ^2^ Department of Cardiology, Sanjay Gandhi Post Graduate Institute of Medical Sciences, Lucknow, India; ^3^ Amity Institute of Microbial Technology, Amity University, Noida, India

**Keywords:** outbreak, multi-drug resistant, *K. pneumoniae*, antimicrobial stewardship, hospital infection control

## Abstract

Infections caused by multi-drug resistant *Klebsiella pneumoniae* are a leading cause of mortality and morbidity among hospitalized patients. In neonatal intensive care units (NICU), blood stream infections by *K. pneumoniae* are one of the most common nosocomial infections leading to poor clinical outcomes and prolonged hospital stays. Here, we describe an outbreak of multi-drug resistant *K. pneumoniae* among neonates admitted at the NICU of a large tertiary care hospital in India. The outbreak involved 5 out of 7 neonates admitted in the NICU. The antibiotic sensitivity profiles revealed that all *K. pneumoniae* isolates were multi-drug resistant including carbapenems and colistin. The isolates belonged to three different sequence types namely, ST-11, ST-16 and ST-101. The isolates harboured carbapenemase genes, mainly *bla*_NDM-1_, *bla*_NDM-5_ and *bla*_OXA-232_ besides extended-spectrum β-lactamases however the colistin resistance gene *mcr-1*, *mcr-2 and mcr-3* could not be detected. Extensive environmental screening of the ward and healthcare personnel led to the isolation of *K. pneumoniae* ST101 from filtered incubator water, harboring *bla*_NDM-5_, *bla*_OXA-232_ and ESBL genes (*bla*_CTX-M_) but was negative for the *mcr* genes. Strict infection control measures were applied and the outbreak was contained. This study emphasizes that early detection of such high-risk clones of multi-drug resistant isolates, surveillance and proper infection control practices are crucial to prevent outbreaks and further spread into the community.

## Introduction

*Klebsiella pneumoniae*, a Gram negative bacterium, is a member of the order *Enterobacterales.* It is a dreaded pathogen owing to its potential for carbapenemase production and harbouring multiple resistance genes. The evolution and rapid dissemination of carbapenem resistant *K. pneumoniae* is a significant problem worldwide as it is responsible for high mortality and morbidity in hospitalized and immuno-compromised patients (Couto et al., 2007; Gorrie et al., 2017). These characteristics make it a ‘difficult to treat’ pathogen. Outbreaks in hospitals due to resistant isolates of *Klebsiella pneumoniae* have been described across the world. Emergence of nosocomial multi-drug resistant *K. pneumoniae* infections can be attributed to acquisition and evolution of new resistance genes, use of invasive devices, immuno-compromised state, inappropriate use of antibiotics and inadequate surveillance system (Ayukekbong et al., 2017). The transmission may occur from patient to patient or through the hands of healthcare staff. Neonatal sepsis is one the leading cause of mortality and morbidity in hospitalized neonates (Shah et al., 2015). In neonatal intensive care unit (NICUs), bloodstream infections are one of the most common nosocomial infections and neonatal sepsis accounts for 28% to 50% of all such cases with high prevalence of *K. pneumoniae* (Hassuna et al., 2020). A recent study has reported the emergence of colistin resistant carbapenemase producing *K. pneumoniae* belonging to diverse sequence types in 14 neonates admitted in NICU of tertiary care hospital in India (Sharma et al., 2022). There is paucity of reports on blood stream infections among neonates attributed to multi-drug resistant *K. pneumoniae* in India. The aim of the present study was to characterize the *Klebsiella pneumoniae* isolates from an outbreak in NICU at a tertiary care hospital in India. The pathogen was isolated from 5 neonates over a period of 2 weeks. We also investigated for the possible sources of dissemination and isolated *Klebsiella pneumoniae* from environment of the same ward. The study also reports about the infection control measures enforced to contain the outbreak.

## Materials and methods

We conducted an outbreak investigation which was suspected to have involved 5 out of 7 neonates admitted in a 7 bedded Neonatal Intensive Care Unit (NICU) at Sanjay Gandhi Post Graduate Institute of Medical Sciences, a 900 bed tertiary care referral hospital in North India, during the last week of June till second week of July, 2021.

### Bacterial strains

The blood samples from the neonates were received at our laboratory for routine bacterial culture and sensitivity testing. A total of 5 multi-drug resistant *K. pneumoniae* isolates were recovered. All the isolates were identified by conventional biochemical tests and later confirmed by automated MALDI-TOF-MS system (BioMérieux, Marcy l’Étoile, France).

### Demographic and clinical data collection

All the demographic and clinical data of the patients were collected from the hospital information system of the institute.

### Antimicrobial susceptibility testing

The antibiotic susceptibility pattern and minimum inhibitory concentration (MIC) was determined by liquid broth microdilution method. The results were interpreted according to Clinical and Laboratory Standards Institute (CLSI) guidelines (CLSI, 2021) except for colistin for which European Committee on Antimicrobial Susceptibility Testing (EUCAST) breakpoints were followed (EUCAST, 2021).

### Investigation of resistance mechanism

The resistance mechanisms in the 5 *K. pneumoniae* isolates were analyzed using polymerase chain reaction to detect the genes encoding carbapenemases (*bla*_NDM_, *bla*_KPC_, *bla*_OXA-48_, *bla*_IMP_, *bla*_VIM_), ESBLs (*bla*_CTX-M_), other class A β-lactamases (*bla*_SHV_, *bla*_TEM_) and colistin resistance genes *mcr-1*, *mcr-2* and *mcr-3* using previously described methods and primers (Singh et al., 2018; Singh et al., 2021). Following isolates from our previous studies were used as positive controls: *K. pneumoniae* CR*kp*21 and CR*kp*22 for *bla*_NDM_, *bla*_OXA-48_, *bla*_IMP_, *bla*_VIM_, *bla*_CTX-M_, *bla*_SHV_, *bla*_TEM_ (Singh et al., 2021) while *K. pneumoniae* CRL3 for *mcr* gene (Singh et al., 2018). Positive control for *bla*_KPC_ gene was not available. *E. coli* DH5α was used as a negative control. The details of the primers used in the study are provided in the **Table S1**.

### Clonal diversity and multi-locus sequence typing

Clonal diversity was examined by pulse field gel electrophoresis (PFGE) using *XbaI* (Invitrogen Inc., USA) digested DNA of 5 clinical isolates and one environmental isolate of *K. pneumoniae* according to a previously described method (Qin et al., 2014). The results were analyzed using the criteria laid down by Tenover et al. (1995). Banding patterns were analyzed and dendrogram was generated using BioNumerics software version 7.6 (Applied-Maths, Sint-Martens-Latem, Belgium).

Multi-locus sequence typing (MLST) of 5 *K. pneumoniae* isolates was performed as described previously (Diancourt et al., 2005). The seven housekeeping genes were amplified and sequenced. The sequence type (ST) was assigned by determining the allele number for each of the housekeeping genes using the database maintained by Pasteur Institute at http://bigsdb.pasteur.fr/klebsiella/klebsiella.html/.

### Environmental screening

Screening cultures (swabs) were obtained from different sites in NICU like entrance of room, incubator, oxygen connectors, IV stand, 10% dextrose, normal saline, reverse osmosis water, ringer lactate. The samples were inoculated onto Blood agar and MacConkey agar and incubated for 16-18 hours aerobically. Samples were also inoculated in the Robertson cooked meat broth for 16-18 hours. Sub-cultures were done on blood agar and MacConkey agar after 48 hours. Air samples were also taken by the air sampler for air bio-load monitoring.

### Infection control measures

In order to control outbreaks and hospital acquired infections, we have a hospital infection control committee comprising of hospital administrators, microbiologists, physicians, nurses, technical staff and sanitation workers. During the outbreak we applied strict infection control measures including a) temporary evacuation of the infected ICU with transfer of all patient to the alternative ICU area; b) the empty infected ICU was cleaned by fogging and sodium hypochlorite; c) use of shared medical equipment were stopped; d) sanitation workers were educated about cleaning processes, cleaning protocol were established which included use of disposable cloths for each area using sodium hypochlorite solution kept in closed container to prevent evaporation and replacing it in every 24 hours; e) hand hygiene inspection and observation of infection control nurses and resident doctors were increased, educational sessions of standard precautions and hand hygiene practices were carried out by infection control physicians and nurses; f) environmental screening of bacterial cultures from different sites and hands of hospital personnel were continued;

Besides the NICU and its staff, the infection control measures were implemented in other areas of the hospital especially other ICUs and critical care units. The adherence to infection control measures in the NICU was approximately 70% before the outbreak but it was enhanced to 100% to contain the outbreak and was continued for the next one month, to prevent any further outbreaks.

### NCBI data submission

The nucleotide sequences of the *bla*_NDM_ and *bla*_OXA-232_ genes were submitted in the NCBI GenBank database under accession numbers OL544134-OL544138 and OK351259-OK351263 respectively.

## Results

Out of 5 neonates with blood stream infections, 3 were preterm and 2 were term babies. The demographic details of the patients are given in **Table 1**. All the 5 samples were positive for *Klebsiella pneumoniae* isolates (KPNIC1, KPNIC3, KPNIC4, KPNIC5 and KPNIC6) as confirmed by MALDI-TOF-MS. Among the environmental samples, filtered incubator water was positive for multi-drug resistant *K. pneumoniae* (KPNIC2). Antibiotic sensitivity testing revealed that all the isolates, including the environmental isolate, were resistant to first generation cephalosporins, carbapenems, aminoglycosides and colistin except KPNIC6 which was sensitive to ciprofloxacin (**Table 2**).

**Table 1:** Patient demographics and; details of *K. pneumoniae* isolates, sequence types and antibiotic resistance genes.

Patient S. No.	Age at the time of admission	Gender	Co-morbidities	Days before onset of symptoms	Isolate ID	Source	Antibiotic resistance genes	MLST Sequence type (ST)	Duration of hospital stay	Clinical Outcomes
1.	1 day	Female	Preterm birth	26 days	KPNIC4	Blood	*bla*_NDM-1_, *bla*_OXA-232_, *bla*_SHV,_ *bla*_TEM,_ *bla*_CTX-M_	ST16	47days	Dead
2.	16 days	Female	Portosystemic shunts	7 days	KPNIC6	Blood	*bla*_SHV_	ST101	63 days	Normal discharge
3.	6 days	Male	Acute Respiratory Distress Syndrome (ARDS)	7 days	KPNIC3	Blood	*bla*_NDM-5_, *bla*_OXA-232_, *bla*_SHV_, *bla*_TEM_	ST101	25 days	Normal discharge
4.	1day	Female	Preterm birth	2 days	KPNIC5	Blood	*bla*_NDM-1_, *bla*_OXA-232_, *bla*_VIM_, *bla*_SHV_, *bla*_CTX-M_	ST11	50 days	Normal discharge
5.	15 days	Male	Preterm birth	2 days	KPNIC1	Blood	*bla*_NDM-5_, *bla*_OXA-232_, *bla*_SHV_, *bla*_TEM_	ST16	62 days	Normal discharge

**Table 2:** Antimicrobial susceptibility and minimum inhibitory concentration (MIC) table of *K. pneumoniae* isolates.

Organism/No. of Isolates	Antibiotics (Range tested, µg/ml)	CLSI (2021) MIC breakpoint ≤S/≥R	Isolates with MIC (µg/ml)					
			KPNIC1	KPNIC2	KPNIC3	KPNIC4	KPNIC5	KPNIC6
*Klebsiella pneumoniae* (n= 6)	Imipenem (1-512)	≤1/≥4	128	128	128	128	256	64
	Meropenem (1-512)	≤1/≥4	128	128	128	128	128	128
	Tobramycin (1-512)	≤2/>4	≥512	256	≥512	256	256	16
	Aztreonam (1-512)	≤4/≥16	128	256	128	128	128	128
	Colistin (1-512)	≤1/≥4	≥512	≥512	≥512	≥512	≥512	≥512
	Ciprofloxacin (1-512)	≤4/≥16	32	≥512	32	≥512	128	8
	Ceftazidime (1-512)	≤4/≥16	≥512	≥512	≥512	≥512	≥512	256
	Cefotaxime (1-512)	≤0.25/≥1	≥512	512	512	512	256	128

**Figure 1:**
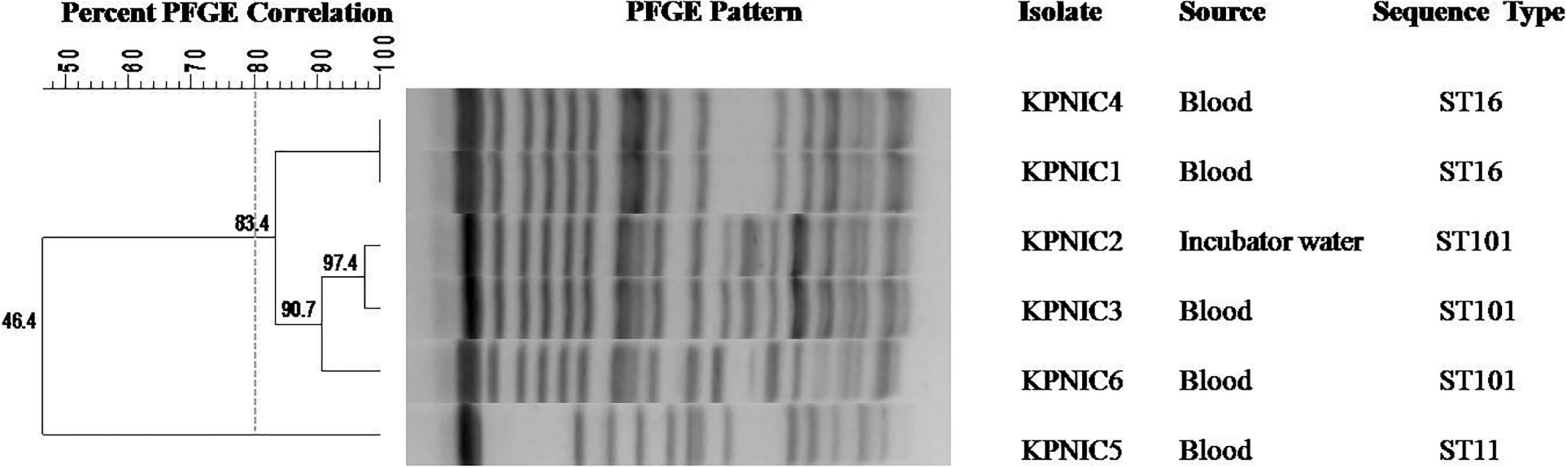
Dendrogram generated by Bionumerics software, showing results of the cluster analysis on the basis of PFGE fingerprinting of five clinical and one environmental isolate of *Klebsiella pneumoniae*. Similarity analysis was performed using Dice coefficient (optimization 1.5%, tolerance 1%) and clustering was done by the unweighted-pair group method (UPGMA). A similarity coefficient of 80% was chosen for cluster definition and grey dotted line shows the delineation line. The degree of similarity is shown in the scale.

Molecular screening of carbapenemase genes revealed that *bla*_NDM-5_ was present in 2 isolates (KPNIC1 and KPNIC3) while *bla*_NDM-1_ was present in 2 isolates (KPNIC4 and KPNIC5). The gene *bla*_OXA-232_ (*bla*_OXA-48_ like variant) co-existed with *bla*_NDM-1_ and *bla*_NDM-5_ in all these 4 isolates. Among other carbapenemases, *bla*_IMP_ gene was absent in all while *bla*_VIM_ was present in one isolate (KPNIC5). Screening of other β-lactamases revealed that *bla*_SHV_ gene was present in all isolates which is intrinsic to *K. pneumoniae* while *bla*_TEM_ was present in 3 isolates (KPNIC1, 3, 4). Among ESBLs, *bla*_CTX_-_M_ co-existed with *bla*_NDM-1_ in 2 isolates (KPNIC4 and KPNIC5) and was absent in others. Colistin resistance genes *mcr*-1,-2 and-3 were absent in all isolates. Other variants of the *mcr* gene and the chromosomal mutations were not investigated. The environmental isolate of *K. pneumoniae* was positive for *bla*_NDM-5_, *bla*_OXA-232_, *bla*_SHV_, *bla*_TEM_, and *bla*_CTX-M_ but negative for *mcr*. Clonal lineage profiling by MLST revealed that out of 5 clinical isolates 2 each belonged to ST101 and ST16 respectively while 1 isolate belonged to ST11. The environmental isolate belonged to ST101 (**Table 1**). Clonal diversity analysis using PFGE revealed that the two ST101 clinical isolates (KPNIC3 and KPNIC6), the ST101 environmental isolate (KPNIC2) and the two ST16 clinical isolates (KPNIC1 and KPNIC4) were closely related to each other while the ST11 clinical isolate (KPNIC5) belonged to a different pulsotype. The two clinical isolates and the environmental isolate belonging to ST101 clustered together with >90% similarity while the ST16 isolates displayed >83% similarity with the ST101 cluster (**Figure 1**).

In order to evaluate the effects of infection control measures and decrease in prevalence of *K. pneumoniae* in the NICU, environmental screening was repeated from different sites in the ward, and from the hospital personnel; and it was observed that after 48 hours of aerobic incubation all the samples were sterile.

## Discussion

Hospital acquired infections are one of the leading cause of prolonged hospital stays and mortality among admitted patients. Studies have revealed that multidrug resistant *Klebsiella pneumoniae* is responsible for 18% to 31% of all nosocomial infections. Mortality due to *K. pneumoniae* is reported to be 30% to 54% in ICUs (Couto et al., 2007; Tao et al., 2014; Stefaniuk et al., 2016). We report the investigation of an outbreak due to *K. pneumoniae* in an NICU of a tertiary care hospital in the most populated state of north India. The outbreak involved 5 out of 7 neonates admitted in the NICU. Among the 5 neonates, 3 were preterm babies while the other 2 had portosystemic shunt and ARDS respectively at the time of admission. It is likely that the patients obtained *K. pneumoniae* isolates in the hospital as all of them tested positive 48 hours post admission. Hospital acquired infections are those infections acquired in hospital that first appear 48 hour or more after admission (Revelas, 2012). In Europe and US, there are several reports of outbreaks with carbapenem resistant *K. pneumoniae* and majority of them are associated with *bla*_KPC_. In Asian countries, such outbreaks have been found to be associated with MBLs (Ballot et al., 2019). In India, *bla*_NDM_ and *bla*_OXA-48_ and its variants have often been found to be vehicles of swift dissemination of such isolates (Bakthavatchalam et al., 2016; Sharma et al., 2022). In our study, *bla*_NDM-1_ and *bla*_NDM-5_ co-existed with *bla*_OXA-232_ along with *bla*_SHV_, *bla*_TEM_ and *bla*_CTX-M_ in majority of the isolates. The isolates were resistant to colistin but we could not detect *mcr* gene in any of the isolates possibly because the isolates harboured the chromosomal mutations leading to colistin resistance which we did not analyze. In a recent study from our centre we have reported *K. pneumoniae* ST16 harboring mutations in chromosomal gene *mgrB* (Singh et al., 2021). Multi-locus sequence typing results revealed that the 5 *K. pneumoniae* isolates belonged to 3 different sequence types, ST101, ST16 and ST11. The isolate obtained from the incubator water belonged to ST101. PFGE is considered to be an important tool for molecular epidemiology of bacterial isolates. PFGE data indicated that both clinical and environmental isolates belonging to ST101 and the clinical isolates belonging to ST16 were closely related to each other according to the criteria laid down by Tenover et al. (1995). The ST16 belongs to clonal group CG17/20, ST101 to CG43 while ST11 belongs to CG258 (Wyres et al., 2020). The CG258 consisting of ST11, ST258 and ST512 is a global problem clonal group and is responsible for approximately 60% of all global outbreaks (Navon-Venezia et al., 2017). ST11 is a paraphyletic group of CG258 while ST258 arose from ST11-like ancestor following genomic recombination events (Wyres and Holt, 2016). Studies have reported that ST16 and ST101 belong to the sub-lineages of ST258 believed to have evolved because of large genomic recombination events (Wyres and Holt, 2016; Cherak et al., 2021). Recent reports suggest that ST101 is an international clone and is a potential candidate for becoming high risk multi-drug resistant *K. pneumoniae* in future (Loconsole et al., 2020). In India, a study from south India has reported *K. pneumoniae* ST101 carrying *bla*_KPC-2_ in bloodstream infection (Shankar et al., 2018). In case of our study the gene *bla*_KPC_ was not detected indicating that the plasmid carrying the gene was not present in any of the isolates. The *K. pneumoniae* ST16 is an internationally spread clone but studies have reported that it shows high variability in terms of antimicrobial resistome content thereby indicating that different variants of the clone are circulating in different countries (Espinal et al., 2019). A study performed on 344 *K. pneumoniae* isolates collected from 8 different centers in India has reported 3% prevalence of ST16 with variable genomic profile (Nagaraj et al., 2021). In our study also, 2 isolates KPNIC1 and KPNIC4 belonging to ST16 had different resistome profiles which is similar to the pattern observed in other studies. To the best of our knowledge, this is the second report describing presence of ST11from India. Initially, reported from China, a recent retrospective study which included 8 centers from India reported ST11 in 1.3% of *Klebsiella pneumoniae* clinical isolates. These strains are hypervirulent and have been described in association with spread of KPC, especially in China and Taiwan (Nagaraj et al., 2021).

Hospital acquired infections are a major cause of morbidity and mortality in admitted patients. Overcrowded healthcare facilities especially in low and low-middle income countries are hot-spots for dissemination of multi-drug resistant bacteria. Infections due to colistin resistant carbapenemase producing Gram negative bacterial pathogens are a menace as there are limited options available to treat such infections. Transmission of such multi-drug resistant organisms can be controlled by strict infection control measures and proper hand-hygiene practices. In the current outbreak, we tracked the source of environmental isolate to the jars used for storing incubator water in the NICU. The staffs responsible for autoclaving of jars were informed and corrective measures taken which led to the containment of outbreak.

## Conclusion

This study investigates an outbreak of colistin resistant carbapenemase (*bla*_NDM_ and *bla*_OXA-232_) producing *K. pneumoniae* involving neonates admitted in NICU at a large tertiary care hospital in north India. The outbreak had a serious bearing on the clinical outcomes leading to mortality and prolonged hospital stays of the neonates. Molecular epidemiology of the clinical isolates revealed presence of sequence types ST16, ST101 and ST11 but the PFGE pattern indicated that the ST16 and ST101 isolates were closely related. While carbapenemase associated ST16 and ST101 are internationally spread clones and have been associated with outbreaks of *K. pneumoniae* in various countries including India, ST11 is not a prevalent sequence type in India. One major limitation of our study was lack of whole genome sequencing of the isolates that provides detailed information about the resistome profiles and also helps to understand the dissemination pattern of the high-risk clones. In addition to the transmissible *mcr* gene, chromosomal mutations in *mgrB*, *phoP*/*phoQ*, *pmrA* and *pmrB* genes are responsible for colistin resistance but we did not analyze the alternative mechanisms and it was a limitation of our study. Further, we could track the source of the isolates belonging to ST101 to the incubator water but the source of ST11 could not be traced. Furthermore, we could not isolate ST16 from the environmental samples and after surveillance of the healthcare personnel although it was found to be closely related to the ST101 obtained from the incubator water in our study, indicating that more rigorous surveillance and environmental screening should have been performed. However, owing to the close relatedness of five out six isolates in the current outbreak we believe that the incubator water was the source of infection. Further, after taking corrective measures for proper autoclaving of the jar by the concerned staff the outbreak was contained. Though infection control measures were not robust in our set up but our surveillance actions spread more awareness among the staff. Early detection of such high-risk clones of multi-drug resistant isolates, surveillance and proper infection control practices are crucial to prevent outbreaks and further spread into the community.

## Data availability statement

The datasets presented in this study can be found in online repositories. The names of the repository/repositories and accession number(s) can be found below: The nucleotide sequences of the *bla*_NDM_ and *bla*_OXA-232_ genes were submitted in the NCBI GenBank database under accession numbers OL544134-OL544138 and OK351259-OK351263 respectively.

## Ethics statement

The studies involving human participants were reviewed and approved by Institutional Ethics Committee, Sanjay Gandhi Post Graduate Institute of Medical Sciences, Lucknow, India. Written informed consent for participation was not required for this study in accordance with the national legislation and the institutional requirements.

## Author contributions

CS, AP and NT conceived and designed the study. AP and NT wrote the manuscript. CS critically revised the manuscript. All authors carried out the experiments and collected the data. All authors contributed to the article and approved the submitted version.
