# Supplementary material for: Outbreak of colistin resistant, carbapenemase (bla NDM, bla OXA-232) producing Klebsiella pneumoniae causing blood stream infection among neonates at a tertiary care hospital in India
Source: Front Cell Infect Microbiol. 2023 Feb 1;13:1051020. doi: 10.3389/fcimb.2023.1051020 (PMC9929527; doi:10.3389/fcimb.2023.1051020)
Supplement: Supplementary file 1 [file Table_1.docx]

Table S1: List of primers used in the study

| Gene | Forward primer (5’→3’) | Reverse primer (5’→3’) | Amplicon size  ( base pairs) | References |
| --- | --- | --- | --- | --- |
| *mcr-1* | CGGTCAGTCCGTTTGTTC | CTTGGTCGGTCTGTAGGG | 309 | Singh et al , 2021 |
| *mcr-2* | TGTTGCTTGTGCCGATTGGA | AGATGGTATTGTTGGTTGCTG | 567 |  |
| *mcr-3* | TTGGCACTGTATTTTGCATTT | TTAACGAAATTGGCTGGAACA | 542 |  |
| *bla*_KPC_ | TGTCACTGTATCGCCGTC | GTCAGTGCTCTACAGAAAACC | 1011 |  |
| *bla*_OXA-48_ | TATATTGCATTAAGCAAGGG | CACACAAATACGCGCTAACC | 848 |  |
| *bla*_NDM_ | CACCT CATGTTTGAATTCGCC | CTCTGTCACATCGAAATCGC | 984 |  |
| *bla*_VIM_ | GATGGTGTTTGGTCGCAT | CGAATGCGCAGCACCAG | 390 |  |
| *bla*_IMP_ | GGAATAGAGTGGCTTAAYTCTC | CCAAACYACTASGTTATCT | 188 |  |
| *bla*_SHV_ | GGGTTATTCTTATTTGTCGCT | TAGCGTTGCCAGTGCTCG | 929 |  |
| *bla*_TEM_ | AAAATTCTTGAACG | TTACCAAATGCTTAATCA | 1080 |  |
| *bla*_CTX-M_ | TTTGCGATGTGCAGTACCAGTAA | CGATATCGTTGGTGGTGCCATA | 544 |  |
